# Examining the role of employability as a mediator in the relationship between psychological capital and objective career success amongst occupational psychology professionals

**DOI:** 10.3389/fpsyg.2022.958226

**Published:** 2022-12-14

**Authors:** Vicki Elsey, Beatrice Van der Heijden, Michael A. Smith, Mark Moss

**Affiliations:** ^1^Applied Work Psychology Group, Department of Psychology, Northumbria University, Newcastle upon Tyne, United Kingdom; ^2^Institute for Management Research, Radboud University, Nijmegen, Netherlands; ^3^School of Management, Open Universiteit Nederland, Heerlen, Netherlands; ^4^Faculty of Economics and Business Administration, Ghent University, Ghent, Belgium; ^5^Hubei Business School, Hubei University, Wuhan, China; ^6^Kingston Business School, Kingston University, London, United Kingdom

**Keywords:** employability, psychological capital, objective career success, sustainable careers, occupational psychology

## Abstract

Employability is core to our understanding of career sustainability, and at an individual level, identifying the personal resources that support employability in the achievement of career success is warranted. This study builds on the conservation of resources theory, examining the role of employability as a mediator in the relationship between psychological capital and objective career success. To test our hypotheses, we utilised a context-specific practitioner sample of 135 individuals with UK-accredited occupational psychology qualifications. Employability was conceptualised using the competence-based model, underpinned by occupational expertise. Psychological capital and employability were measured using self-report questionnaires, whilst career success was determined via gross annual salary and practitioner status, ensuring objective measures of this outcome variable. Structural equation modelling identified that the relationship between psychological capital and objective career success was fully mediated by employability. These novel findings have important theoretical and practical implications for the role of psychological capital as a personal resource in achieving career success via its influence on employability.

## Introduction

Maximising employees’ career success (CS) lies at the core of current thinking on “sustainable careers” (see Van der Heijden and De Vos, 2015; Van der Heijden et al., 2020). The sustainable careers notion is purported to be a specific form of human sustainability, where individuals are creating, testing, and maintaining their ability to adapt (Holling, 2001), and is receiving increasing research consideration (e.g., Veld et al., 2016; Anseel, 2017), influencing recent conceptual papers (De Vos et al., 2017, 2020). The COVID-19 pandemic has accelerated awareness of the need to protect one’s career sustainability, testing individual and organisational resilience and adaptability in a constantly changing world (Hite and McDonald, 2020). COVID-19 implies a “career shock”, referring to a highly disruptive and extraordinary event (Akkermans et al., 2020, p. 2), which may lead individuals to question and change their career direction. It is important to develop an understanding of what resources may support individuals through their careers as these are becoming more complex, less predictable, and require more individual agency (Akkermans and Kubasch, 2017). Empirical work in the field is at the initial stages of development (Van der Heijden et al., 2020).

Important to sustainable careers is the concept of employability (Veld et al., 2016), particularly in a post-COVID world (Zhou et al., 2022). Two broad definitions explain employability as an input (antecedent) or output (outcome). Input theories refer to knowledge, skills, abilities, and other characteristics (KSAOs) that assist individuals in finding employment, measuring employability indirectly via dispositions (Fugate et al., 2004) or competencies (Van der Heijden et al., 2018). Output theories or “self-perceived” employability measure employability directly via an internal assessment of one’s ability to find and retain work (Rothwell and Arnold, 2007; Berntson et al., 2008; Vanhercke et al., 2014). Input approaches are yet to be fully investigated in working populations, with the majority of employability research focusing on self-perceived employability. Ultimately, this focus has limited our understanding of the role of employability for career outcomes, which is urgently needed for developing interventions and sustainability.

Personal resources in the form of self-esteem, optimism, career adaptability, and so on have gained recent research interest demonstrating their relationship with career outcomes, including career success, both objective and subjective (Haenggli and Hirschi, 2020), and link to the conservation of resources (COR) theory (Hobfoll, 1989). The current research aims to build on COR and investigates an empirical model of how psychological capital (PsyCap), being a personal resource and an individual malleable state, enhances perceptions of input employability and also objective CS (OCS).

Psychological capital (PsyCap) is a higher-order construct that includes optimism, self-efficacy, resilience, and hope (Luthans et al., 2007b). As a personal resource, and thus an element of human capital, it is theoretically proposed as an important precursor for employability (Fugate et al., 2004). For applied researchers and practitioners, the state-like, malleable nature of PsyCap makes it a popular concept, with an evidence base demonstrating its benefits. Examples include improved individual performance (Luthans et al., 2007a), happiness and dedication (Larson and Luthans, 2006; Luthans et al., 2008b), and job search behaviour (Hulshof et al., 2020). In addition, a small amount of research has also begun to link PsyCap to career outcomes such as career commitment (Gan and Cheng, 2021); yet, this research line is emerging, and more scholarly work is needed given its complexity (Kauffeld and Spurk, 2021). Recently, research has sought to identify the antecedents of PsyCap, summarising that there are indeed many more research opportunities to be explored around this construct (Vilariño del Castillo and Lopez-Zafra, 2022).

Psychological capital (PsyCap) represents people-based advantages that enable individuals to harness improvements across workplace behaviours (Newman et al., 2014, p. 120) and, therefore, we posit that it comprises a valuable resource for one’s sustainable employability (see also Tomlinson et al., 2017). Empirical work examining the link between PsyCap and employability is limited. Research suggests that PsyCap relates to job insecurity; a relationship that is partially mediated by self-perceived employability (Chiesa et al., 2018). In addition, PsyCap is related to employability perceptions in the unemployed (Ngoma and Ntale, 2016), indicating that PsyCap can enhance the perceptions of employability (Chiesa et al., 2018, p. 7). More widely reported are the relationships between career constructs, such as employability and career success, on the one hand, and individual components of PsyCap, on the other hand. For instance, self-efficacy has been investigated as a predictor for salary and subjective career success (Abele and Spurk, 2009; Dacre-Pool and Qualter, 2013; Ahmed et al., 2019), optimism as an adaptive psychological resource to support career success (Lounsbury et al., 2003; Spurk et al., 2015, p. 413; Spurk et al., 2019), and resilience as a predictor of job search behaviours in the unemployed (Fleig-Palmer et al., 2009), of subjective career success (Ahmad et al., 2019) and of sustainable employability measured via vitality in Dutch police officers (Semeijn et al., 2019). Hope has received least attention, although aspects of hope (such as goal setting) have been linked to greater OCS (Abele and Spurk, 2009), proactive career behaviours in students (Clements and Kamau, 2018), and to employability (Liu et al., 2020), and goal setting appears to be an important component of career self-management (Hirschi et al., 2018).

It is important to note that not all research suggests relationships between PsyCap constructs, on the one hand, and employability and career success, on the other hand. Perhaps, the most noteworthy is self-efficacy, with contradictory research in this area. Whilst researchers generally agree that self-efficacy is related to but different from employability (Fugate et al., 2004; Van der Heijde and Van der Heijden, 2006; Berntson et al., 2008), there have been inconsistencies in how both the constructs of self-efficacy and employability have been measured, leading to alternative conclusions. These include employability and career success leading to self-efficacy due to an accumulation of positive experiences (Berntson et al., 2008), not the other way around, and additionally that there are reciprocal benefits.

However, PsyCap is a higher-order factor, and Luthans et al. (2006) suggested that (PsyCap) is greater than the sum of its parts (human and social capital) (p. 21), and these authors argue that we need more insight into both this higher-order factor and its individual components. Interestingly, in the previous work in this field, there are contradictions between whether PsyCap (or its components) can directly predict OCS or whether this relationship is enhanced by some other mechanism, such as employability [e.g., as in Chiesa et al. (2018)], and whilst we believe the research suggests direct relationships, we intend to understand this further through our empirical work.

As far as we are aware, no previous studies have considered the predictive power of PsyCap on employability using the competence-based approach developed by Van der Heijde and Van der Heijden (2006). Employability competencies are important indicators of one’s potential and are argued as a strong antecedent of individual career success (Van der Heijde and Van der Heijden, 2006; Van der Heijden et al., 2009). The core notion postulates that skilled use and, if needed, further development of competencies will lead to greater perceptions of employability (Wittekind et al., 2010; Vanhercke et al., 2014). Underpinned by an earlier suggestion that up-to-date occupational expertise (i.e. domain-specific knowledge owned by the individual) is a requirement for one’s employability sustainability (Van der Heijden, 2002). Occupational expertise is accompanied by four competencies: (1) anticipation and optimisation: working creatively and planning for and adjusting to future challenges; (2) personal flexibility: flexing to internal and external job market changes; (3) corporate sense: partaking in activities outside specific job roles, for example, sharing experiences with professional networks; and (4) balance: achieving compromises between individual and organisational goals (Van der Heijde and Van der Heijden, 2006). It is worth knowing that there is research to suggest that perceived employability (i.e. the output perspective) is not related to OCS (Bargsted et al., 2021). This urges us to be very explicit about the specific conceptualisation of employability that we use in scholarly work, as it is obvious that there are differences in the way one measures employability, depending on the discipline wherein it is studied, the focal stakeholders, key responsibilities, and key outcomes, amongst others (Fugate et al., 2021). PsyCap, as an individual resource, and competence-based employability provide a rich research foundation on which to understand career sustainability in professional groups, who often have to harness their own resources and build occupational expertise in the pursuit of career success.

Some empirical work investigates employability amongst academic staff, hospital and care staff, and manufacturing staff in the Dutch workforce (Van der Klink et al., 2014; Veth et al., 2015; Van der Heijden et al., 2016; Van der Heijden and Spurk, 2019); yet, research on working UK populations is scarce. In addition, we respond to calls to understand the context (Scalise et al., 2019; Weng and Zhu, 2020), in career research by concentrating on a homogeneous sample of UK-based occupational psychology (OP) professionals, where “occupational expertise” defines career and leads, therefore, to the adoption of the competence-based model of employability (Van der Heijde and Van der Heijden, 2006).

Occupational psychology (OP) is concerned with human behaviour in the workplace, also referred to as industrial and organisational (IO), work or business psychology. OP professionals careers are complex, often self-managed, individualised, and characterised by roles that may not have the title “occupational psychologist” (Elsey et al., 2020) and, therefore, comprise a challenging path for individual practitioners. Whilst career literature theoretically discusses the individual nature of career management and employability as an individual responsibility (Zheltoukhova and Baczor, 2016), it offers little in the way of practical suggestions to support individuals. OP employability and professional sustainability were a key priority for the profession [Division of Occupational Psychology (DOP) Strategic Plan, 2016-2020] and were the focus of the DOP (2021) Virtual Conference. Using this niche professional group enables us to develop our understanding through a microscope and not use a “broad brush” approach to defining careers, which is often criticised in the career literature (Abele and Wiese, 2008; Forrier et al., 2018). Developing contextual understanding is the first step to gain more knowledge about career similarities and differences across professions, herewith enabling more targeted interventions and a more robust evidence base on which to intervene to support careers. It is hoped that this research encourages replication across many professional groups within psychology and more broadly.

**FIGURE 1:**
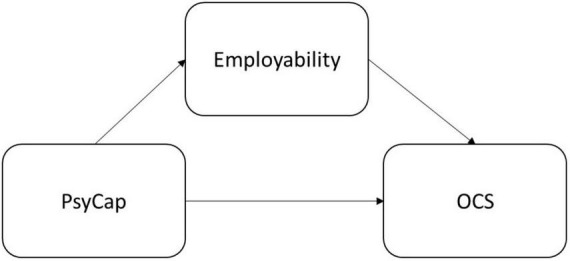
The hypothesised (partial) mediation model of objective career success enhancement.

The present study, therefore, aims to investigate whether PsyCap is associated with a competence-based conceptualisation of employability. We further aim to examine whether the relationship between PsyCap and OCS is mediated by employability as conceptualised in Figure 1. We hypothesise that:

**Hypothesis 1):** There will be a positive correlation between PsyCap and employability in occupational psychology professionals.

**Hypothesis 2):** Employability will partially mediate the relationship between PsyCap and OCS in occupational psychology professionals (see Figure 1).

## Materials and methods

### Participants and procedure

Ethical approval to conduct the research was granted following scrutiny from the Department of Psychology Ethics Committee at the University of Northumbria, UK. The research was advertised on social networking sites (including Facebook, Twitter, and LinkedIn), targeting groups with OP professionals and advertising at the Division of Occupational Psychology Annual Conference. The principal investigator emailed university alumni, accredited OP UK Programme Directors, and their professional network, asking them to share the survey with their contacts. Thus, adopting a snowballing approach (Atkinson and Flint, 2004) to maximise participation. Interested participants could follow a link to the study questionnaire on SurveyMonkey.^1^ Participation took an average of 30 min and all participants were debriefed fully on the aims of the research.

**FIGURE 2:**
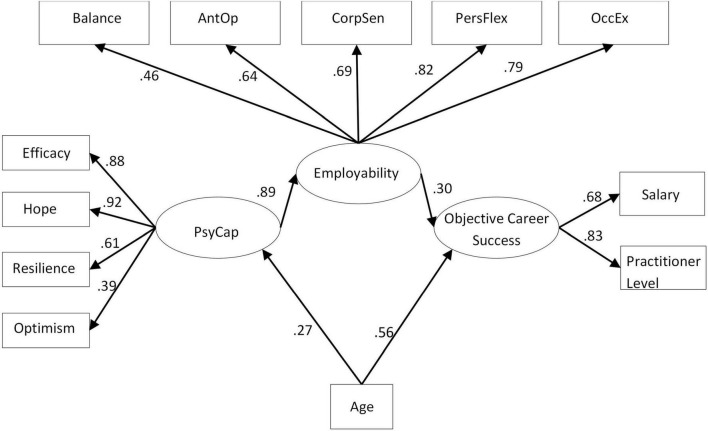
Significant structural paths for alternative model with employability as a full mediator between PsyCap and objective career success.

An a priori power analysis suggested that for our specified model, with three latent variables and 12 (parcelled) observed variables (Hall et al., 1999; Little et al., 2002; see Figure 2, for parcelling), a minimum of 119 participants would be needed to detect a medium effect at 0.8 power. The final sample comprised 135 participants (30 men and 105 women; 71.4% of returned questionnaires; responses were removed where participants completed consent and demographic questions but no other questions). All participants were required to have achieved Graduate Basis for Chartered (GBC) status with the BPS and completed an MSc in OP (The Stage One Qualification in OP, UK-based and accredited by the British Psychological Society), therefore, controlling for qualifications which afforded equivalence in education.

A total of 118 participants disclosed their age, ranging between 22 and 59 years (M = 34.29, SD = 8.38). At the same time, 119 participants disclosed information about professional membership; 31% (n = 42) were Chartered Psychologists and/or Registered Practitioners with the HCPC (Health and Care Professions Council), 28% (n = 37) were contemplating embarking on the Stage Two OP Qualification to become HCPC Registered, 10% (n = 14) were Trainee OPs (i.e. enrolled on the Stage Two Qualification), 19% (n = 26) stated that were not pursuing the Stage Two Qualification or had no professional body membership. Finally, all participants worked in OP-related roles, such as consultant, psychologist (business, work, organisational, or occupational), learning and development, human resources, lecturer, company director, or organisational development.

### Measures

To reduce common-method bias, Podsakoff et al. (2003) suggested procedure was followed. This included informing participants of anonymity and confidentiality and stressing that there were no correct or incorrect responses. Finally, participants were informed that all data would be analysed in a collective, not individual, way. The risk of acquiescence, that is, answering positively on all scales (see McCrae, 2018), was addressed by the different scale anchors used in the questionnaires. The study was also confirmed to be independent of any organisation or professional body. Permission to use the measures was sought from the authors or publisher, and scales were selected based on psychometric properties, relevance for the target audience, and the theoretical justification outlined in the introduction.

#### Demographic variables

Age is a contributing factor in career perceptions, with research suggesting that employability is associated with chronological age (Fugate et al., 2004; Van der Heijde and Van der Heijden, 2005; McQuaid, 2006; Clarke and Patrickson, 2008; Van der Heijden et al., 2009; Le Blanc et al., 2017), and that age is positively correlated with salary (Ng et al., 2005). Due to prior research suggesting a relationship between age and the study variables, we have included this variable in our study.

#### Psychological capital

The 24-item PsyCap Questionnaire (PCQ) (Luthans et al., 2007a) was utilised, measuring four aspects of PsyCap: optimism (e.g., I always look on the bright side of things regarding my job), self-efficacy (e.g., I feel confident presenting information to a group of colleagues), resilience (e.g., I feel I can handle many things at a time in this job), and hope (e.g., I can think of many ways to reach my current work goals). Each construct was rated by six statements on a scale where 1 represents strongly disagree and 6 represents strongly agree. Previous research indicates individual construct coefficient alphas vary between 0.66 and 0.89 (ibid.) and, in the current research, between 0.77 and 0.87.

#### Employability

Employability was measured via Van der Heijde and Van der Heijden’s (2006) competence-based employability tool, which included 47 items relating to the five dimensions of employability: occupational expertise (e.g., I consider myself competent to engage in in-depth, specialist discussions in my job domain), anticipation, and optimisation (e.g., In formulating my career goals I take account of external market demand), personal flexibility (e.g., How easily would you say you can adapt to changes in your workplace?), corporate sense (e.g., In my organisation I take part in forming a common vision of values and goals), and balance (e.g., My work and private life are easily balanced). Rating was on a 6-point scale (1 representing the lower end). Previous research indicates Cronbach’s alpha for occupational expertise between 0.82 and 0.96, for anticipation and optimisation of between 0.67 and 0.91, personal flexibility between 0.68 and 0.89, for corporate sense of 0.83 to 0.92 and balance ranging from 0.82 to 0.96 (ibid), and in the current sample between 0.79 and 0.93.

#### Objective career success

Gross annual salary and practitioner level (determined by consultation with practitioners in the field) were utilised as measures of OCS (1 = entry/trainee and junior practitioner, 2 = practitioner, 3 = senior practitioner, and 4 = Chief Executive or Director).

### Analysis strategy

We checked for missing data before conducting the analyses, and where appropriate, data imputation was used (replacement by respondent’s mean where single scale items were missing, no more than 10% missing). Data imputation was not conducted on age, gender, current practitioner level, and salary. Confirmatory factor analysis (CFA) using AMOS 26 was carried out to confirm the presence of an appropriate factor structure. Item loadings of less than 0.5 (Chin, 1998) were removed. This led to the removal of a single item from the PsyCap measure and nine items from the employability measure (final versions of the questionnaire and CFA analysis can be provided on request), which were then parcelled (Little et al., 2002) according to their theoretical constructs. To test the measurement and hypothesised model, structural equation modelling (SEM) using AMOS 26 was adopted. Alternative model (AM) testing was utilised on both measurement and structural models. Our measurement model was tested with three latent factors: PsyCap, employability, and OCS. Model fit was determined through fit indices chi-square (χ^2^) and indices less impacted by sample size (see also Fan et al., 1999; and MacCallum and Austin, 2000), including root-mean-square error of approximation (RMSEA), normed chi-square (χ^2^/df), and comparative fit indices (CFI; Byrne, 2001). Values between 1 and 5 on χ^2^/df indicate sufficient model fit, with values lower than 2 being preferred, values close to 1 suggested an improved fit for CFI, and RMSEA ≤ 0.05 indicates good fit and ≤ 0.08 is acceptable (Browne and Cudeck, 1993).

## Results

### Preliminary analysis of descriptive statistics and correlations

Good internal consistencies were observed by coefficient alphas for all PsyCap measures and employability measures (see Table 1). We observed significant positive correlations amongst all aspects of PsyCap and the five dimensions of Employability. Salary and practitioner level correlated positively with occupational expertise, Personal flexibility, and corporate sense and with the self-efficacy and hope subscales of PsyCap. Salary and optimism were positively correlated. Age correlated with salary, practitioner level, occupational expertise, self-efficacy, and hope. None of the study variables were significantly correlated with gender (Table 1).

**TABLE 1:** Descriptive statistics and correlation coefficients of the study variables (diagonal/parentheses represent Cronbach’s alpha).

	M	SD	1	2	3	4	5.1.	5.2.	5.3.	5.4.	5.5.	6.1.	6.2.	6.3.	6.4.
1. Gender^1^	1.78	0.42	–												
2. Age (years) (N = 118)	34.29	8.38	–0.07	–											
3. Gross Annual Salary	£37,023	£18,665	–0.12	0.44**	–										
4. Practitioner Level^2^	2.13	0.80	–0.02	0.53**	0.57**	–									
5. Employability															
5.1. Occupational Expertise (OE)	4.91	0.61	–0.10	0.29**	0.33**	0.39**	(0.93)								
5.2. Anticipation and Optimisation (AO)	4.33	0.85	–0.05	0.10	0.09	0.11	0.42**	(0.81)							
5.3. Personal Flexibility (PF)	4.46	0.57	–0.11	0.16	0.23*	0.26*	0.65**	0.52**	(0.79)						
5.4. Corporate Sense (CS)	4.59	0.88	–0.01	0.08	0.12	0.23**	0.54**	0.47**	0.58**	(0.85)					
5.5. Balance (B)	4.05	0.82	0.05	0.16	0.17*	0.15	0.37**	0.37**	0.39**	0.28**	(0.89)				
6. PsyCap															
6.1. Self-Efficacy (SE)	4.82	0.74	–0.06	0.30**	0.34**	0.37**	0.69**	0.46**	0.58**	0.54**	0.30**	(0.87)			
6.2. Optimism (Op)	4.34	0.73	–0.06	0.12	0.17*	0.16	0.41**	0.41**	0.45**	0.34**	0.36**	0.37**	(0.77)		
6.3. Hope (Ho)	4.71	0.70	–0.04	0.19*	0.23**	0.30**	0.58**	0.59**	0.68**	0.58**	0.36**	0.63**	0.54**	(0.86)	
6.4. Resilience (Re)	4.73	0.65	–0.08	0.15	0.09	0.15	0.44**	0.35**	0.48**	0.27**	0.34**	0.36**	0.54**	0.42**	(0.80)

*p < 0.05. **p < 0.01. 1. Gender coded 1 = male, 2 = female; 2. Practitioner level 1 = junior/entry, 2 = practitioner, 3 = senior practitioner, 4 = Director/Chief Executive.

### The measurement model: A preliminary analysis

The measurement model yielded an acceptable model fit 𝒳^2^ (40) = 56.356, p = 0.045, χ^2^/df = 1.409, CFI = 0.973, RMSEA = 0.055. All estimates from the observed variables to the second-order latent variables were significant at the p < 0.001 level (employability variables: balance = 0.47, anticipation and optimisation = 0.65, corporate sense = 0.69, personal flexibility = 0.82, and occupational expertise = 0.78; PsyCap variables: optimism = 0.56, self-efficacy = 0.77, resilience: = 52, and hope = 0.82; OCS variables: salary = 0.69; practitioner level = 0.82). We observed better fit for the three-factor model over the one-factor model where PsyCap, employability, and objective success factors were grouped together [𝒳^2^ (44) = 112.614, p = 0.001, CMIN/df = 2.559 CFI = 0.886, RMSEA = 0.108].

### Testing hypothesis 2: Partial mediation between psychological capital and objective career success—The structural model

Next, we tested our structural model, where employability was a partial mediator between PsyCap and OCS (hypothesis 2). Fit indices are presented in Table 2. We found support for hypothesis 2 [𝒳^2^ (44) = 59.916, p = 0.193, CMIN/df = 1.181, CFI = 0.987, RMSEA = 0.037]. We also tested an alternative model (AM) where employability was included as a full mediator between PsyCap and OCS (Table 2). AM presented slightly better fit to the data [𝒳^2^ (45) = 52.078, p = 0.218, CMIN/df = 1.157, CFI = 0.989, RMSEA = 0.034]. In addition, AM demonstrated statistically significant relationships between the model’s paths which were not the case in the hypothesised model. Therefore, AM had the most appropriate fit to the data that could be theoretically explained and was accepted in favour of the hypothesised model.

**TABLE 2:** Measurement model, hypothesised model, and alternative model fit.

Model	χ^2^	df	p	CMIN /df	CFI	RMSEA
Measurement (correlational) model	56.356	40	0.045	1.409	0.973	0.055
Hypothesised baseline model partial mediation	51.916	4	0.193	1.181	0.987	0.037
Alternative Model Full Mediation	52.078	45	0.218	1.157	0.989	0.034

It is clear from Figure 2 (accepted alternative model, showing all significant paths) that PsyCap significantly and positively predicted employability (β = 0.89,*p* < 0.001). Those individuals with greater PsyCap demonstrated higher perceptions of their employability competencies. In addition, employability significantly and positively predicted OCS (β =  0.30,*p* <  0.01) in that higher employability led to increased OCS. There were also significant paths between age and objective success, as well as between age and PsyCap (β =  0.56,*p* <  0.001andβ =  0.27,*p* = 0.002, respectively). Contrary to the partial mediation model predicted in hypothesis 2, these data support a full mediation of employability between PsyCap and OCS, and instead, the alternative model of full mediation was supported.

## Discussion

The present study aimed to understand the role that PsyCap and employability played in the OCS of individuals working in OP. In doing so, we responded to calls for context-specific career research (Scalise et al., 2019; Weng and Zhu, 2020) by focusing on a professional group of occupational psychology professionals, provided an important contribution to extant employability literature, also adopting the competence-based conceptualisation of employability (Van der Heijde and Van der Heijden, 2006), not previously explored in a UK context, but relevant for our hypotheses. As predicted, in H1, we observed a positive relationship between PsyCap and employability, and despite predicting that we would observe a partial mediation between the study variables (H2), we found evidence to support a fully mediated model.

The first hypothesis was supported by the significant positive correlations between the four components of PsyCap and the five components of employability; in fact, all correlations were significant. This was anticipated based on the previous literature suggesting that PsyCap is a potential indicator of employability (Williams et al., 2016) but builds upon a heavy emphasis on self-efficacy in employability literature above other PsyCap constructs (Fugate et al., 2004; Berntson et al., 2008; Dacre-Pool and Qualter, 2013). Strong correlations between *self-efficacy* and *occupational expertise* suggest that those individuals who possess confidence also report greater perceptions of their technical and specialist knowledge. This finding supports Bandura’s original definition of self-efficacy, indicating that the best way to build self-efficacy is through mastery experiences (Bandura, 1982) and suggests that in occupational psychology professionals an important step in building occupational expertise could be through experiences. As a correlational result, we do not know whether this could also work the other way around (i.e. occupational expertise builds self-efficacy) or perhaps there are also reciprocal benefits. This finding is worthy of further in-depth investigation. In addition, *Hope* correlated strongly with *Personal Flexibility* supporting Goal Setting Theory (Locke et al., 1981) in making future goals that can enhance career adaptability and planning. This finding relates to research indicating that training university students in goal setting can support their longer-term employability (Clements and Kamau, 2018) and extends it by indicating that, in fact, setting goals can increase one’s ability to adapt to the job market, which is perhaps counter-intuitive. However, it could be possible that in this group of practitioners, people are setting flexible rather than rigid goals, which support them in their pursuit of career success. In fact, previous research suggests that hope supports proactive career behaviour and identification of potential future challenges (Hirschi, 2014) and that hope can support individuals to retain an optimistic focus, linked to the exploration of opportunity, creativity, and taking risks in successful entrepreneurs (Tang, 2020). Again, further investigation due to the correlational nature of the study would be warranted.

With reference to hypothesis 2, we anticipated that employability would be a partial mediator between PsyCap and OCS. The best-fitting model, however, was fully mediated by employability. This suggests that the positive state alone does not directly lead to OCS, measured by salary and practitioner level. Rather, a positive state is needed to protect and further enlarge one’s competencies, which then results in OCS, and this adds prediction over and above age alone (Van der Heijde and Van der Heijden, 2006; Van der Heijden et al., 2009). What was different in our research was that age and employability were not represented by a significant path in our model. In fact, age only correlated with the occupational expertise component of employability (see Table 1). This finding is worthy of further investigation in this population (and other professional groups) to identify whether there is a “tipping point” or whether there are greater nuances in the age employability relationship, depending on context, contrary to previous research (De Lange et al., 2021). The future research is also needed incorporating different conceptualisations of age, over and above just calendar age [see, for instance, the categorisation by Sterns and Doverspike (1989) into chronological age, functional or performance-based age, psychosocial or subjective age, organisational age, and the concept of lifespan age] (cf. De Lange et al., 2021).

What is interesting about our findings is that full mediation was the best-fitting model, and not partial mediation, despite prior research leading to the development of this hypothesis (e.g., Chiesa et al., 2018). These findings are correlational and, thus, must be treated with caution. Speculatively, it may be possible to improve employability by focusing on investments in PsyCap and through an increase in employability one’s OCS could be enhanced. This is different from the research by Chiesa et al. (2018), who identified that employability perceptions partially mediated the relationship between PsyCap and, in their case, job insecurity as the outcome. The fact that our employability measure was competence-based might be part of the explanation. Furthermore, longitudinal research would be needed to understand whether this could be replicated. In addition, Bargsted et al. (2021) found that employability related to OCS but suggested that OCS was less in the control of the individual and, therefore, not easily enhanced by personal resources. The latter supports our findings that the personal resource of PsyCap did not directly impact OCS but indirectly *via* employability. Indeed, research indicates that resilience can help individuals to cope with career shock (Akkermans et al., 2020), and it is entirely possible that the relationship found in our research between PsyCap and employability is the mechanism by which the impact of career shock is managed. To safely conclude and to better understand the underlying mechanism requires further research.

Psychological capital’s malleability (Luthans et al., 2006, 2008a,b, 2010; Luthans and Youssef-Morgan, 2017), coupled with the relationships observed in our study, indicates that interventions focused on developing PsyCap may enhance employability and, in turn, improve one’s OCS. These findings build upon our current understanding of the role of employability in the relationship between personal resources and OCS, adding to the emerging evidence-base on the importance of PsyCap in career and employability outcomes through a lens of COR. This research also identifies how resources can foster improved perceptions of employability and, in turn, objective career success, adding to the growing amount of research around predictors of both employability (Fugate et al., 2021) and career success (Kauffeld and Spurk, 2021), and their role in promoting sustainable careers (Ngo et al., 2013; Newman et al., 2014; Cenciotti et al., 2017; Alessandri et al., 2018; Van der Heijden et al., 2020).

### Implications for future research, policy, and practice

Our research points to the potential benefits of PsyCap in enhancing employability when using a competence-based employability framework strategically chosen to fit with the professional group under investigation (after Vanhercke et al., 2014). PsyCap interventions could occur at multiple points in the career journey of OP practitioners to build their resources and support them in managing their careers (Akkermans and Kubasch, 2017). In addition, the competence-based model of employability promotes occupational expertise as an element of human capital essential to career success. In this practitioner group of occupational psychologists, it would seem that occupational expertise, alongside the other elements of employability, is important to fostering objective career success. Higher education providers might consider how the educational environment can be utilised as a way of building the resources necessary to support a sustainable career. This is over and above the current focus on self-efficacy (Dacre Pool and Sewell, 2007; Williams et al., 2016) as the cumulative impact of PsyCap may prove fruitful to the development of employability and, in turn, OCS, and in addition to our findings suggest further consideration of hope, which is often overlooked in the literature. Furthermore, the route to independent practice for OP graduates is long and challenging (Elsey et al., 2020), so the development of PsyCap throughout one’s career could build personal resource caravans (Hobfoll, 1988, 2011), which, in turn, enhance employability and OCS, a consideration for individuals, the professional body, and employers alike. Importantly, we must understand how to increase PsyCap as a way to foster sustainable careers. However, our research suggests that objective career success can only be improved indirectly through employability and not directly by investments in PsyCap, and thus we must develop a further understanding of what employability means in professional groups and how this resource is harnessed by individuals.

### Limitations and directions for future research (split section)

We recognise that the strength of our study was in the establishment of a pattern of relationships between psychological capital, employability, and objective career success in a homogenous sample; however, we recognise its limitations. By design, the study was self-report and cross-sectional. The future research would benefit from a multi-method approach (e.g., multi-source ratings and supervisor ratings) as in previous employability research (e.g., Van der Heijden et al., 2009, 2016; McAbee and Connelly, 2016). Where obtaining supervisor ratings is difficult (e.g., self-employed), asking clients or colleagues, who form part of the individuals’ network could be used as alternative.

Second, we measured a limited number of variables determined by the study’s hypotheses. Due to the complexity of careers and potential factors which could impact objective success and employability, the future work should control for fixed traits, such as personality, and investigate other “capitals”, for example, social, movement, and identity (Eby et al., 2003; Fugate et al., 2004; Ng and Feldman, 2014; Forrier et al., 2015; Tomlinson, 2017; Clarke, 2018). In addition, our focus on two markers of objective career success only (gross annual salary and practitioner level) may have failed to capture some of the complexities associated with career success. We excluded other variables seen in the literature, such as a number of promotions due to the involvement of self-employed practitioners (Elsey et al., 2020) and the reality of boundaryless careers, where lateral moves rather than hierarchical (i.e. promotions) are common (DeFillippi and Arthur, 1994). We also deliberately included an objective career outcome measure to avoid common-method bias and following research suggesting that happy individuals are generally more positive in research (Hogan et al., 2013). A future emphasis on subjective career success would enable us to understand individual perceptions, which could be based on factors over and above salary and level, potentially including opportunities for growth and development, herewith responding to the positivist emphasis traditionally observed (Dries, 2011; Spurk et al., 2019). Furthermore, the research could utilise the indicators as conceptualised in the notion of sustainable careers (i.e. health, happiness, and productivity; Van der Heijden et al., 2020) in addition to objective measures. Understanding the lived experiences of individuals in this profession to build on this research and on the work by Elsey et al. (2020) would further support our understanding of exactly how resources are utilised in support of career outcomes alongside quantitative research.

Finally, the sample size of 135, was an acceptable cut-off for conducting SEM and within the *a priori* parameters for a medium effect (Boomsma, 1985; Wolf et al., 2013), but it meant that we could not utilise full disaggregation models and instead operated at the parcelled level, a convention used in many studies (such as Little et al., 2002; Abele and Spurk, 2009; De Hauw and De Vos, 2010; Van der Heijden and Bakker, 2011). Nonetheless, parcelling does not allow us to fully appreciate the distinct contribution of individual variables, which could lead to model misspecification, particularly in multi-dimensional frameworks (Little et al., 2002). In the future, SEM should be performed on item-level data, as well as utilising newer, shorter versions of questionnaires, such as the 22-item short-form competence-based measure of employability (Van der Heijden et al., 2018).

## Conclusion

In conclusion, our research adds to the emerging evidence-base on PsyCap as a personal resource in careers but importantly looks in further depth at how it can support career outcomes *via* employability. Our findings indicate that PsyCap can enhance perceptions of employability which, in turn, can lead to greater OCS and that the relationship between PsyCap and OCS is full, not partially (as hypothesised), mediated by employability. In addition, our research points to some interesting relationships between components of PsyCap and employability, such as hope and personal flexibility, which are fruitful avenues for future research. For practitioners, understanding the factors that can enhance career outcomes, such as OCS, provides useful information on where to intervene when supporting and developing those in occupational psychology professions. We call for future research to consider our limitations and suggestions and to apply similar research methodology to a range of professional careers, accounting for the occupational expertise element of employability and thus building a more nuanced understanding of modern-day, sustainable careers.

## Data availability statement

The raw data supporting the conclusions of this article will be made available by the authors, without undue reservation.

## Ethics statement

The study and protocol were reviewed and approved by Northumbria University, Department of Psychology Ethics Committee. All participants read a participant briefing and provided consent online to participate in the study.

## Author contributions

VE and MM designed the study. VE collected and analysed the data with support from MM and MS and wrote the first draft. BV and MS wrote and edited specified sections of the manuscript. BV supported VE in translating the results and linking back to theory and recent empirical research. All authors were involved in the production of the final manuscript, reading, and approved the final submitted version.

## Footnotes

1
www.surveymonkey.com

## References

[B1] Abele A. E. Spurk D. (2009). The longitudinal impact of self-efficacy and career goals on objective and subjective career success. *J. Vocat. Behav.* 74 53–62. 10.1016/j.jvb.2008.10.005

[B2] Abele A. E. Wiese B. S. (2008). The nomological network of self-management strategies and career success. *J. Occup. Organ. Psychol.* 81 733–749. 10.1348/096317907X256726

[B3] Ahmad B. Latif S. Bilal A. R. Hai M. (2019). The mediating role of career resilience on the relationship between career competency and career success. *Asia-Pac. J. Bus. Adm*. 11 209–231. 10.1108/APJBA-04-2019-0079

[B4] Ahmed H. Nawaz S. Rasheed M. I. (2019). Self-efficacy, Self-esteem, and Career Success: The Role of Perceived Employability. *J. Manag. Sci.* 6 18–32. 10.20547/jms.2014.1906202

[B5] Akkermans J. Kubasch S. (2017). #Trending topics in careers: A review and future research agenda. *Career Dev. Int*. 22 586–627. 10.1108/CDI-08-2017-0143

[B6] Akkermans J. Richardson J. Kraimer M. L. (2020). The Covid-19 crisis as a career shock: Implications for careers and vocational behavior. *J. Vocat. Behav.* 119:103434. 10.1016/j.jvb.2020.103434 32390655 PMC7205633

[B7] Alessandri G. Consiglio C. Luthans F. Borgogni L. (2018). Testing a dynamic model of the impact of psychological capital on work engagement and job performance. *Career Dev. Int*. 23 33–47. 10.1108/CDI-11-2016-0210

[B8] Anseel F. (2017). Agile learning strategies for sustainable careers: A review and integrated model of feedback-seeking behavior and reflection. *Curr. Opin. Environ. Sustain.* 28 51–57. 10.1016/j.cosust.2017.07.001

[B9] Atkinson R. Flint J. (2004). “Snowball sampling,” in *The SAGE Encyclopaedia of Social Science Research Methods*, eds Bryman A. Lewis-Beck M. Liao T. (Washington DC: Sage Publications), 1043–1044.

[B10] Bandura A. (1982). Self-efficacy mechanism in human agency. *Am. Psychol.* 37:122. 10.1037/0003-066X.37.2.122

[B11] Bargsted M. Yeves J. Merino C. Venegas-Muggli J. I. (2021). Career success is not always an outcome: Its mediating role between competence employability model and perceived employability. *Career Dev. Int*. 26 119–139. 10.1108/CDI-06-2020-014

[B12] Berntson E. Näswall K. Sverke M. (2008). Investigating the relationship between employability and self-efficacy: A cross-lagged analysis. *Eur. J. Work Organ. Psychol.* 17 413–425. 10.1080/13594320801969699

[B13] Boomsma A. (1985). Nonconvergence, improper solutions, and starting values in LISREL maximum likelihood estimation. *Psychometrika* 50 229–242. 10.1007/BF02294248

[B14] Browne M. W. Cudeck R. (1993). “Alternative ways of assessing model fit,” in *Testing Structural Equation Models*, eds Bollen K. Long J. (Washington DC: Sage Publications), 111–135.

[B15] Byrne B. M. (2001). *Structural Equation Modeling With AMOS: Basic Concepts, Applications, and Programming.* London: Psychology Press.

[B16] Cenciotti R. Alessandri G. Borgogni L. (2017). Psychological capital and career success over time: The mediating role of job crafting. *J. Leadersh. Organ. Stud.* 24 372–384. 10.1177/1548051816680558

[B17] Chiesa R. Fazi L. Guglielmi D. Mariani M. G. (2018). Enhancing substainability: Psychological capital, perceived employability, and job insecurity in different work contract conditions. *Sustainability* 10:2475. 10.3390/su10072475

[B18] Chin W. W. (1998). “The partial least squares approach to structural equation modeling,” in *Modern methods for business research*, ed. Marcoulides G. A. (Hillsdale, NJ: Lawrence Erlbaum Associates Publishers), 295–336.

[B19] Clarke M. (2018). Rethinking graduate employability: The role of capital, individual attributes and context. *Stud. High. Educ.* 43 1923–1937. 10.1080/03075079.2017.1294152

[B20] Clarke M. Patrickson M. (2008). The new covenant of employability. *Empl. Relat.* 30 121–141. 10.1108/01425450810843320

[B21] Clements A. J. Kamau C. (2018). Understanding students’ motivation towards proactive career behaviours through goal-setting theory and the job demands–resources model. *Stud. High. Educ.* 43 2279–2293. 10.1080/03075079.2017.1326022

[B22] Dacre Pool L. Sewell P. (2007). The key to employability: Developing a practical model of graduate employability. *Educ. Train.* 49 277–289. 10.1108/00400910710754435

[B23] Dacre-Pool L. Qualter P. (2013). Emotional self-efficacy, graduate employability, and career satisfaction: Testing the associations. *Aust. J. Psychol.* 66 214–223. 10.1111/ajpy.12023

[B24] De Hauw S. De Vos A. (2010). Millennials’ Career Perspective and Psychological Contract Expectations: Does the Recession Lead to Lowered Expectations? *J. Bus. Psychol.* 25 293–302. 10.1007/s10869-010-9162-9

[B25] De Lange A. H. Van der Heijden B. Van Vuuren T. Furunes T. De Lange C. Dikkers J. (2021). Employable as we age? A systematic review of relationships between age operationalizations and employability. *Front. Psychol.* 11:605684. 10.3389/fpsyg.2020.605684 33613362 PMC7893083

[B26] De Vos A. Dujardin J. M. Gielens T. Meyers C. (2017). *Developing Sustainable Careers Across the Lifespan.* Cham: Springer International.

[B27] De Vos A. Van der Heijden B. I. J. M. Akkermans J. (2020). Sustainable careers: Towards a conceptual model. *J. Vocat. Behav.* 117:103196. 10.1016/j.jvb.2018.06.011

[B28] DeFillippi R. J. Arthur M. B. (1994). The boundaryless career: A competency-based perspective. *J. Organ. Behav.* 15 307–324. 10.1002/job.4030150403

[B29] Division of Occupational Psychology (DOP) Strategic Plan (2016-2020). The *British Psychological Society: Division of Occupational Psychology*. Available online at: https://www.bps.org.uk/member-microsites/division-occupational-psychology (accessed July 1, 2020).

[B30] DOP (2021). “School of psychology & neuroscience,” in *Proceedings of the division of occupational psychology (DOP) virtual conference.*

[B31] Dries N. (2011). The meaning of career success. *Career Dev. Int*. 16 364–384. 10.1108/13620431111158788

[B32] Eby L. T. Butts M. Lockwood A. (2003). Predictors of Success in the Era of the Boundaryless Career. *J. Organ. Behav.* 24 689–708. 10.1002/job.214

[B33] Elsey V. (2016). *The Career of an Occupational Psychology Graduate: Employment, Employability and Identity*, Ph.D thesis, London: Northumbria University.

[B34] Elsey V. Thompson N. Sillence E. Longstaff L. Moss M. (2020). Becoming a professional: The five pillars of identification in Occupational Psychology in the UK. *EWOP Practice* 13 42–67.

[B35] Fan X. Thompson B. Wang L. (1999). Effects of sample size, estimation methods, and model specification on structural equation modeling fit indexes. *Struct. Equ. Modeling* 6 56–83. 10.1080/10705519909540119

[B36] Fleig-Palmer M. M. Luthans K. W. Mandernach B. J. (2009). Successful reemployment through resiliency development. *J. Career Dev.* 35 228–247. 10.1177/0894845308327271

[B37] Forrier A. De Cuyper N. Akkermans J. (2018). The winner takes it all, the loser has to fall: Provoking the agency perspective in employability research. *Hum. Resour. Manag. J.* 28 511–523. 10.1111/1748-8583.12206

[B38] Forrier A. Verbruggen M. De Cuyper N. (2015). Integrating different notions of employability in a dynamic chain: The relationship between job transitions, movement capital and perceived employability. *J. Vocat. Behav.* 89 56–64. 10.1016/j.jvb.2015.04.007

[B39] Fugate M. Kinicki A. J. Ashforth B. E. (2004). Employability: A psycho-social construct, its dimensions, and applications. *J. Vocat. Behav.* 65 14–38. 10.1016/j.jvb.2003.10.005

[B40] Fugate M. Van der Heijden B. De Vos A. Forrier A. De Cuyper N. (2021). Is what’s past prologue? A review and agenda for contemporary employability research. *Acad. Manag. Ann.* 15 266–298. 10.5465/annals.2018.0171

[B41] Gan Y. Cheng L. (2021). Psychological capital and career commitment among Chinese urban preschool teachers: The mediating and moderating effects of subjective wellbeing. *Front. Psychol.* 12:3003. 10.3389/fpsyg.2021.509107 34366945 PMC8339257

[B42] Haenggli M. Hirschi A. (2020). Career adaptability and career success in the context of a broader career resources framework. *J. Vocat. Behav.* 119:103414. 10.1016/j.jvb.2020.103414

[B43] Hall R. J. Snell A. F. Foust M. S. (1999). Item parceling strategies in SEM: Investigating the subtle effects of unmodeled secondary constructs. *Organ. Res. Methods* 2 233–256. 10.1177/109442819923002

[B44] Hirschi A. (2014). Hope as a resource for self-directed career management: Investigating mediating effects on proactive career behaviors and life and job satisfaction. *J. Happiness Stud.* 15 1495–1512. 10.1007/s10902-013-9488-x

[B45] Hirschi A. Nagy N. Baumeler F. Johnston C. S. Spurk D. (2018). Assessing key predictors of career success: Development and validation of the career resources questionnaire. *J. Career Assess.* 26 338–358. 10.1177/106907271769558

[B46] Hite L. M. McDonald K. S. (2020). Careers after COVID-19: Challenges and changes. *Hum. Resour. Dev. Int.* 23 427–437. 10.1080/13678868.2020.1779576

[B47] Hobfoll S. E. (1988). *The Ecology of Stress.* Milton Park: Taylor and Francis.

[B48] Hobfoll S. E. (1989). Conservation of resources: A new attempt at conceptualizing stress. *Am. Psychol.* 44 513–524. 10.1037/0003-066X.44.3.513 2648906

[B49] Hobfoll S. E. (2011). Conservation of resource caravans and engaged settings. *J. Occup. Organ. Psychol.* 84 116–122. 10.1111/j.2044-8325.2010.02016.x

[B50] Hogan R. Chamorro-Premuzic T. Kaiser R. B. (2013). Employability and career success: Bridging the gap between theory and reality. *Ind. Organ. Psychol.* 6 3–16. 10.1111/iops.12001

[B51] Holling C. S. (2001). Understanding the complexity of economic, ecological, and social systems. *Ecosystems* 4 390–405. 10.1007/s10021-001-0101-5

[B52] Hulshof I. L. Demerouti E. Le Blanc P. M. (2020). A job search demands-resources intervention among the unemployed: Effects on well-being, job search behavior and reemployment chances. *J. Occup. Health Psychol.* 25 17–31. 10.1037/ocp0000167 31478707

[B53] Kauffeld S. Spurk D. (2021). Why Does Psychological Capital Foster Subjective and Objective Career Success? The Mediating Role of Career-Specific Resources. *J. Career Assessment* 30 285–308. 10.1177/10690727211040053

[B54] Larson M. Luthans F. (2006). Potential added value of psychological capital in predicting work attitudes. *J. Leadersh. Organ. Stud.* 13 75–92. 10.1177/10717919070130020601

[B55] Le Blanc P. M. Van der Heijden B. I. J. M. Van Vuuren T. (2017). I will survive” A construct validation study on the measurement of sustainable employability using different age conceptualizations. *Front. Psychol.* 8:1690. 10.3389/fpsyg.2017.01690 29033875 PMC5627018

[B56] Little T. D. Cunningham W. A. Shahar G. Widaman K. F. (2002). To parcel or not to parcel: Exploring the question, weighing the merits. *Struct. Equ. Modeling* 9 151–173. 10.1207/S15328007SEM0902_1

[B57] Liu X. Peng M. Y. P. Anser M. K. Chong W. L. Lin B. (2020). Key teacher attitudes for sustainable development of student employability by social cognitive career theory: The mediating roles of self-efficacy and problem-based learning. *Front. Psychol.* 11:1945. 10.3389/fpsyg.2020.01945 33117202 PMC7561397

[B58] Locke E. A. Shaw K. N. Saari L. M. Latham G. P. (1981). Goal setting and task performance: 1969–1980. *Psychol. Bull.* 90:125. 10.1037/0033-2909.90.1.125

[B59] Lounsbury J. W. Loveland J. M. Sundstrom E. D. Gibson L. W. Drost A. W. Hamrick F. L. (2003). An investigation of personality traits in relation to career satisfaction. *J. Career Assessment* 11 287–307. 10.1177/1069072703254501

[B60] Luthans F. Avey J. B. Avolio B. J. Norman S. M. Combs G. M. (2006). Psychological capital development: Toward a micro-intervention. *J. Organ. Behav.* 27 387–393. 10.1002/job.373

[B61] Luthans F. Avey J. B. Avolio B. J. Peterson S. J. (2010). The development and resulting performance impact of positive psychological capital. *Hum. Resour. Dev. Q.* 21 41–67. 10.1002/hrdq.20034

[B62] Luthans F. Norman S. M. Avolio B. J. Avey J. B. (2008b). The mediating role of psychological capital in the supportive organizational climate—employee performance relationship. *J. Organ. Behav.* 29 219–238. 10.1002/job.507

[B63] Luthans F. Avey J. B. Patera J. L. (2008a). Experimental analysis of a web-based training intervention to develop positive psychological capital. *Acad. Manag. Learn. Educ.* 7 209–221. 10.5465/amle.2008.32712618

[B64] Luthans F. Youssef C. M. Avolio B. J. (2007b). *Psychological Capital: Developing the Human Competitive Edge.* Oxford: Oxford University Press.

[B65] Luthans F. Avolio B. J. Avey J. B. Norman S. M. (2007a). Positive psychological capital: Measurement and relationship with performance and satisfaction. *Pers. Psychol.* 60 541–572. 10.1111/j.1744-6570.2007.00083.x

[B66] Luthans F. Youssef-Morgan C. M. (2017). Psychological capital: An evidence-based positive approach. *Annu. Rev. Organ. Psychol. Organ. Behav.* 4 339–366. 10.1146/annurev-orgpsych-032516-113324

[B67] MacCallum R. C. Austin J. T. (2000). Applications of structural equation modeling in psychological research. *Annu. Rev. Psychol.* 51 201–226. 10.1146/annurev.psych.51.1.201 10751970

[B68] McAbee S. T. Connelly B. S. (2016). A multi-rater framework for studying personality: The trait-reputation-identity model. *Psychol. Rev.* 123:569. 10.1037/rev0000035 27504526

[B69] McCrae R. R. (2018). Method biases in single-source personality assessments. *Psychol. Assessment* 30 1160–1173. 10.1037/pas0000566 29595291

[B70] McQuaid R. W. (2006). Job search success and employability in local labor markets. *Ann. Reg. Sci.* 40 407–421. 10.1007/s00168-006-0065-7

[B71] Newman A. Ucbasaran D. Zhu F. E. I. Hirst G. (2014). Psychological capital: A review and synthesis. *J. Organ. Behav.* 35 120–138. 10.1002/job.1916

[B72] Ng T. W. Eby L. T. Sorensen K. L. Feldman D. C. (2005). Predictors of objective and subjective career success: A meta-analysis. *Pers. Psychol.* 58 367–408. 10.1111/j.1744-6570.2005.00515.x

[B73] Ng T. W. Feldman D. C. (2014). Subjective career success: A meta-analytic review. *J. Vocat. Behav.* 85 169–179. 10.1016/j.jvb.2014.06.001

[B74] Ngo H. Y. Foley S. Ji M. S. Loi R. (2013). Linking gender role orientation to subjective career success: The mediating role of psychological capital. *J. Career Assessment* 22 290–303. 10.1177/1069072713493984

[B75] Ngoma M. Ntale P. D. (2016). Psychological capital, career identity and graduate employability in Uganda: The mediating role of social capital. *Int. J. Train. Dev.* 20 124–139. 10.1111/ijtd.12073

[B76] Podsakoff P. M. MacKenzie S. B. Lee J. Y. Podsakoff N. P. (2003). Common method biases in behavioral research: A critical review of the literature and recommended remedies. *J. Appl. Psychol.* 88 879–903. 10.1037/0021-9010.88.5.879 14516251

[B77] Rothwell A. Arnold J. (2007). Self-perceived employability: Development and validation of a scale. *Pers. Rev*. 36 23–41. 10.1108/00483480710716704

[B78] Scalise D. Sukumaran N. Merson E. S. Pursell C. Grossman L. Johnson C. (2019). A Qualitative Analysis of Early Career Women’s Adjustment to Work in Professional Psychology: Practitioners’ Reflections. *J. Career Dev.* 46 531–549. 10.1177/0894845318786460

[B79] Semeijn J. H. Caniëls M. C. Kooistra D. (2019). Cross-lagged effects of resilience and indicators of sustainable employability; a study among Dutch police officers. *Policing. Int. J.* 42 961–975. 10.1108/PIJPSM-01-2019-0003

[B80] Spurk D. Hirschi A. Dries N. (2019). Antecedents and outcomes of objective versus subjective career success: Competing perspectives and future directions. *J. Manag.* 45 35–69. 10.1177/0149206318786563

[B81] Spurk D. Kauffeld S. Barthauer L. Heinemann N. S. (2015). Fostering networking behavior, career planning and optimism, and subjective career success: An intervention study. *J. Vocat. Behav.* 87 134–144. 10.1016/j.jvb.2014.12.007

[B82] Sterns H. L. Doverspike D. (1989). “Aging and the retraining and learning process in organizations,” in *Training and Development in Work Organizations*, eds Goldstein I. Katzel R. (San Francisco, CA: Jossey-Bass), 229–332. 10.1016/j.neunet.2020.05.031

[B83] Tang J. J. (2020). Psychological capital and entrepreneurship sustainability. *Front. Psychol.* 11:866. 10.3389/fpsyg.2020.00866 32528347 PMC7248200

[B84] Tomlinson M. (2017). Forms of graduate capital and their relationship to graduate employability. *Educ. Train.* 59 338–352. 10.1108/ET-05-2016-0090

[B85] Tomlinson M. McCafferty H. Fuge H. Wood K. (2017). Resources and Readiness: The graduate capital perspective as a new approach to graduate employability. *J. Natl. Inst. Career Educ. Counselling* 38 28–35. 10.20856/jnicec.3805

[B86] Van der Heijde C. M. Van der Heijden B. I. J. M. (2005). The development and psychometric evaluation of a multi-dimensional measurement instrument of employability—and the impact of aging. *Int. Congr. Ser.* 1280 142–147. 10.1016/j.ics.2005.02.061

[B87] Van der Heijde C. M. Van der Heijden B. I. J. M. (2006). A competence-based and multidimensional operationalization and measurement of employability. *Hum. Resource Manag.* 45 449–476. 10.1002/hrm.20119

[B88] Van der Heijden B. (2002). Prerequisites to guarantee life-long employability. *Pers. Rev.* 31 44–61. 10.1108/00483480210412418

[B89] Van der Heijden B. De Vos A. Akkermans J. Spurk D. Semeijn J. Van der Velde M. (2020). Sustainable careers across the lifespan: Moving the field forward. *J. Vocat. Behav.* 117:103344. 10.1016/j.jvb.2019.103344

[B90] Van der Heijden B. I. J. M. Bakker A. B. (2011). Toward a Mediation Model of Employability Enhancement: A Study of Employee–Supervisor Pairs in the Building Sector. *Career Dev. Q.* 59 232–248. 10.1002/j.2161-0045.2011.tb00066.x

[B91] Van der Heijden B. I. J. M. De Lange A. H. Demerouti E. Van der Heijde C. M. (2009). Age effects on the employability–career success relationship. *J. Vocat. Behav.* 74 156–164. 10.1016/j.jvb.2008.12.009

[B92] Van der Heijden B. I. J. M. De Vos A. (2015). “Sustainable careers: Introductory chapter,” in *Handbook of research on sustainable careers*, eds De Vos A. Van der Heijden B. I. J. M. (Cheltenham: Edward Elgar Publishing), 1–19. 10.4337/9781782547037.00006

[B93] Van der Heijden B. I. J. M. Gorgievski M. J. De Lange A. H. (2016). Learning at the workplace and sustainable employability: A multi-source model moderated by age. *Eur. J. Work Organ. Psychol.* 25 13–30. 10.1080/1359432X.2015.1007130

[B94] Van der Heijden B. I. J. M. Notelaers G. Peters P. Stoffers J. De Lange A. H. Froehlich D. (2018). Development and validation of the short-form employability five-factor instrument. *J. Vocat. Behav.* 106 236–248. 10.1016/j.jvb.2018.02.003

[B95] Van der Heijden B. I. J. M. Spurk D. (2019). Moderating role of LMX and proactive coping in the relationship between learning value of the job and employability enhancement among academic staff employees. *Career Dev. Int.* 24 163–186. 10.1108/CDI-09-2018-0246

[B96] Van der Klink M. Van der Heijden B. I. J. M. Boon J. Williams van Rooij S. (2014). Exploring the contribution of formal and informal learning to academic staff employability A Dutch perspective. *Career Dev. Int.* 19 337–356. 10.1108/CDI-03-2013-0030

[B97] Vanhercke D. De Cuyper N. Peeters E. De Witte H. (2014). Defining perceived employability: A psychological approach. *Pers. Rev.* 43 592–605.

[B98] Veld M. Van der Heijden B. I. J. M. Semeijn J. H. (2016). Home-to-work spillover and employability among university employees. *J. Manag. Psychol.* 31 1280–1296. 10.1108/JMP-09-2015-0347

[B99] Veth K. N. Emans B. Van der Heijden B. I. J. M. Korzilius H. P. L. M. De Lange A. H. (2015). Development (f)or maintenance? An empirical study on the use of and need for HR practices to retain older Workers in Health Care Organizations. *Hum. Resour. Dev. Q.* 26 53–80. 10.1002/hrdq.21200

[B100] Vilariño del Castillo D. Lopez-Zafra E. (2022). Antecedents of psychological capital at work: A systematic review of moderator–Mediator effects and a new integrative proposal. *Eur. Manage. Rev.* 19 154–169. 10.1111/emre.12460

[B101] Weng Q. Zhu L. (2020). Individuals’ Career Growth Within and Across Organizations: A Review and Agenda for Future Research. *J. Career Dev.* 47 239–248. 10.1177/0894845320921951

[B102] Williams S. Dodd L. J. Steele C. Randall R. (2016). A systematic review of current understandings of employability. *J. Educ. Work* 29 877–901. 10.1080/13639080.2015.1102210

[B103] Wittekind A. Raeder S. Grote G. (2010). A longitudinal study of determinants of perceived employability. *J. Organ. Behav.* 31 566–586. 10.1002/job.646

[B104] Wolf E. Harrington K. M. Clark S. L. Miller M. W. (2013). Sample size requirements for structural equation models: An evaluation of power, bias, and solution propriety. *Educ. Psychol. Meas.* 73 913–993. 10.1177/0013164413495237 PMC4334479 25705052

[B105] Zheltoukhova K. Baczor L. (2016). *Attitudes to Employability and Talent.* London: Chartered Institute of Personnel and Development.

[B106] Zhou W. Pan Z. Jin Q. Feng Y. (2022). Impact of Self-Perceived Employability on Sustainable Career Development in Times of COVID-19: Two Mediating Paths. *Sustainability* 14:3753. 10.3390/su14073753

